# Incubation temperature affects the expression of young precocial birds’ fear-related behaviours and neuroendocrine correlates

**DOI:** 10.1038/s41598-018-20319-y

**Published:** 2018-01-30

**Authors:** Aline Bertin, Ludovic Calandreau, Maryse Meurisse, Marion Georgelin, Rupert Palme, Sophie Lumineau, Cécilia Houdelier, Anne-Sophie Darmaillacq, Ludovic Dickel, Violaine Colson, Fabien Cornilleau, Christophe Rat, Joel Delaveau, Cécile Arnould

**Affiliations:** 1 PRC, CNRS, IFCE, INRA, Université de Tours, 37380 Nouzilly, France; 2 Unit of Physiology, Pathophysiology, and Experimental Endocrinology, Department of Biomedical Sciences, University of Veterinary Medicine, 1210 Vienna, Austria; 3 UMR CNRS 6552, Ethos, Université de Rennes1, 35042 Rennes, France; 4 INRA, UR1037 Fish Physiology and Genomics, F-35000 Rennes, France; 5 UE1295 Pôle d’Expérimentation Avicole de Tours, F-37380 Nouzilly, France

## Abstract

The influence of embryonic microclimate on the behavioural development of birds remains unexplored. In this study, we experimentally tested whether chronic exposure to suboptimal temperatures engendered plasticity in the expression of fear-related behaviours and in the expression of the corticotropin-releasing factor in the brains of domestic chicks (*Gallus g. domesticus*). We compared the neurobehavioural phenotypes of a control group of chicks incubated in an optimal thermal environment (37.8 °C) with those of a group of experimental chicks exposed chronically *in ovo* to suboptimal temperatures (27.2 °C for 1 hour twice a day). Chronic exposure to a suboptimal temperature delayed hatching and decreased growth rate and experimental chicks had higher neophobic responses than controls in novel food and novel environment tests. In addition, experimental chicks showed higher expression of corticotropin-releasing factor than did controls in nuclei of the amygdala, a structure involved in the regulation of fear-related behaviours. In this study, we report the first evidence of the strong but underappreciated role of incubation microclimate on the development of birds’ behaviour and its neurobiological correlates.

## Introduction

Among vertebrates, the nongenomic effects of parental environment on the construction of offspring phenotypes are a well-observed phenomenon^1^. These effects drive behavioural plasticity that may constraint or allow offspring to cope better with the conditions experienced by their parents^2,3^. Most studies considering the development of behaviour of avian species have focused on maternal effects mediated by egg quality, egg components, yolk hormones or post-hatching parental care^4^. Far less is known regarding the direct influence of embryos’ thermal environment. This gap in our knowledge is particularly surprising because avian embryos are poikilotherms, and therefore, their development relies entirely on parental incubation behaviour.

Parents of most avian species sit on their eggs and use their bodies to transfer heat to the developing embryos. Parental attentiveness is crucial because the optimal incubation temperature for avian embryos is considered to be narrow, within the range of 35.5 °C and 38.5 °C^5,6^. However, field measurements have shown that embryos are exposed to substantial diel thermal fluctuations when parents leave their nest to forage for instance, and are thus subjected to temperatures above the optimum but more commonly to substantial periodic cooling^5^. Parental incubation behaviour is a period of elevated energy expenditure^7^, and this costly reproductive behaviour is highly flexible because of trade-offs between the self-maintenance and survival of parents and the thermal requirements and survival of developing embryos^8–10^. Depending on a complex balance between the intrinsic condition of parents (metabolic resources, health) and environmental conditions (e.g., food resources, weather and human activities), the microclimate provided to embryos will be close to the optima (reviewed in^11^). In extreme cases, unfavourable conditions may engender egg neglect, when eggs are left unattended by the parents for abnormally extended periods of time^12^. Most notably, the thermal environment profoundly affects the rate of embryonic development. High temperatures decrease incubation periods, whereas cool temperatures slow down embryonic development and delay hatching^12,13^. In addition, embryonic metabolism (*e.g*., heart rate, thermoregulation, oxygen consumption, lipids use) changes substantially to buffer the consequences of unfavourable temperatures (review in^14^).

Contrary to morpho-physiological traits of avian embryos, which have been well described^6,14^, the understanding of the effects of incubation microclimate on the development of chicks’ behavioural traits and subsequent capacities to adapt to their biotic and abiotic environment is very limited. The few studies conducted to date show that suboptimal incubation temperatures impair traits related to fitness such as post-hatching growth rates, survival and reproductive success in adulthood^6^. In reptiles, exposure to low temperatures during incubation may be critical for survival since it was found to alter hatchlings’ capacities to learn the location of a hide^15^. Because birds and reptiles show very similar morpho-physiological responses to suboptimal temperatures during incubation^14^, avian embryos may have also evolved a series of responses encompassing both morpho-physiological and behavioural traits. Nongenomic contributions of parents are considered a key link between environmental conditions and resulting phenotypic outcomes^16^. Currently, nongenomic contributions of parents to behavioural phenotypes have been primarily studied *via* yolk hormones of maternal origin deposited into the egg^17^. We hypothesized that the temperature during incubation would influence the developmental plasticity of young birds’ behaviour.

To test this hypothesis, we examined whether chronic exposure to a suboptimal temperature created conditions for the phenotypes of young birds to be shaped by the hypothetical parental environment. We used an experimental paradigm under controlled thermal conditions. Our subjects were domestic chicks (*Gallus g. domesticus*), because studies of domestic chickens have long provided important insights into the potential importance of incubation temperature for wild birds. In addition, raising precocial chicks without a mother allows us to distinguish the effects on development of the prenatal environment from the effect of parental care after hatching. We compared the neurobehavioural phenotypes of a control group of chicks incubated in a thermal environment empirically considered optimal for hatchability, growth and the quality of hatchlings of layers, with those of a group of experimental chicks exposed repeatedly *in ovo* to suboptimal temperatures. Until day 12 of incubation, both groups of eggs were maintained in the same incubator at the constant optimal temperature of 37.8 °C. From day 12 through to 19, the group of experimental eggs was placed twice a day for 1 hour in a similar incubator with equivalent hygrometry but maintained at 27.2 ± 0.2 °C. During incubation, we recorded heart rates to evaluate the effects of the temperature treatment on embryos. At hatching, we evaluated the expression of the corticotropin-releasing factor in neonate brains in different nuclei of the arcopallium/amygdala, a brain region involved in fear-related behaviours. Birds and mammals’ corticotropin-releasing factor systems play a key role in the control of the hypothalamic-pituitary-adrenal axis and the regulation of fear-related behaviours (like fear of novelty) or social behaviours^18^. The hypothalamic-pituitary-adrenal axis regulates corticosterone plasma levels, the major glucocorticoid hormone in birds. To investigate non-invasively potential differences of adrenocortical activity, we assayed levels of fecal corticosterone metabolites^19^. We characterized the behavioural phenotypes of young chicks maintained under controlled thermal conditions and focused our attention in particular on fear of novelty (neophobia). Indeed, neophobia is widespread in bird populations, and this plastic trait is related to the capacity of animals to adapt to novel food or novel environments^20^. This trait can be cause for concern for bird welfare on farms as it could impair fowl’s capacities to adapt to changes in their feeding regime (feeding transitions)^21^. Neophobia is often thought to play a key role in species’ niche breadth, home range size or dietary choices^22,23^. The dangerous niche hypothesis argues that neophobia protects animals from unknown potential dangers, and that high-risk environments should favor the expression of this plastic trait^20^. Given the acknowledged ecological role of neophobia it is surprising that environmental factors engendering developmental plasticity still remained unexplored. We evaluated social motivation and social discrimination, which are key components of social adaptation for gregarious animals. Chicks’ capacities to solve a locomotor detour problem were assessed. According to the literature^14^, we predicted that the differences in the thermal environment during incubation would alter embryonic development and hatchlings’ growth. We expected the embryonic temperature to be a source of young birds’ subsequent behavioural plasticity. As birds and fish express greater fearfulness (i.e. propensity to express fear responses) following prenatal stress^24,25^, we expected experimental chicks to express higher neophobia and enhanced reactivity to social separation compared with control chicks. As the corticotropin-releasing factor system regulates the expression of birds’ fear related behaviours, we expected the density of corticotropin-releasing factor cells to be higher in brain nuclei of experimental chicks than in those of control chicks.

## Results

### Influence of periodic cooling on embryos’ heart rate and growth

Our experimental embryos reacted to the temperature variations. On all recorded days, experimental embryos’ heart rates (number of beats per minute) decreased significantly during the 1-hour cooling treatment (Table 1), whereas control embryos’ heart rates did not differ significantly (Table 1). Hatching success did not differ significantly between the experimental group and the control group (86 of the 88 fertile eggs in the experimental group hatched and 75 of the 82 control fertile eggs hatched; Chi-square-test, *P* = 0.06). The cooling treatment increased incubation duration significantly (experimental chicks: 21.57 ± 0.03 days; control chicks: 20.94 ± 0.02 days, *F*_*1,159*_ = 173.21, *P* < 0.0001). The cooling treatment significantly altered the growth of experimental chicks compared with control chicks (Table 2; treatment effect: *F*_*1,94*_ = 7.32, *P* = 0.008; sex effect: *F*_*1,94*_ = 31.23, *P* < 0.0001; treatment X sex effect: *F*_*1,94* = _3.28, *P* = 0.85). The mass of chicks did not differ significantly between groups at hatching, but the mass of experimental chicks was significantly lower than that of control chicks at 10 (*F*_*1,1*_ = 5.53, *P* = 0.02) and 16 days after hatching (*F*_*1,1*_ = 9.78, *P* = 0.002), and a non-significant trend was apparent on day 25 (*F*_*1,1*_ = 3.19, *P* = 0.07). Food intake did not differ significantly between experimental and control groups on day 11 (29.58 ± 0.86 vs. 28.58 ± 0.95 g, respectively, *F*_*1,46*_ = 0.61, *P* = 0.44) or 23 (49.96 ± 2.42 vs. 47.04 ± 2.71 g, respectively, *F*_*1,46*_ = 0.001, *P* = 0.98).

**Table 1:** Embryonic heart rates before and after 1 hour of cooling treatment.

Group	Embryonic heart rate (Beats per minute)							
	ED 12		ED 13		ED 14		ED 15	
	before	after	before	after	before	after	before	after
Experimental eggs (n = 12)	246.04 ± 15.79	116.73 ± 11.29*	265.63 ± 12.36	106.75 ± 5.27*	271.87 ± 6.97	102.54 ± 3.88*	282.51 ± 9.89	111.48 ± 5.36*
Wilcoxon tests	*z* = −3.06 *P* = 0.002		*z* = −3.06 *P* = 0.002		*z* = −3.06 *P* = 0.002		*z* = −3.06 *P* = 0.002	
Control eggs (n = 12)	257.58 ± 16.84	261.18 ± 20.34	257.0 ± 14.54	270.94 ± 11.73	279.18 ± 8.98	281.47 ± 9.58	281.13 ± 8.32	284.30 ± 8.96
Wilcoxon tests	*z* = −0.15 *P* = 0.85		*z* = −1.49 *P* = 0.14		*z* = −1.33 *P* = 0.18		*z* = −0.63 *P* = 0.53	

Mean beats per minute ± s.e.m. of experimental and control embryos on embryonic days (ED) 12, 13, 14 and 15 and Wilcoxon tests outcomes (intragroup comparisons). *P < 0.05.

**Table 2:** Mass of chicks.

	Group	Sex	Day 1	Day 10	Day 16	Day 25
**Body mass (g)**	Control chicks	Male	43.68 ± 0.54	94.00 ± 1.60	158.66 ± 3.21	291.40 ± 5.80
		Female	42.13 ± 0.42	89.54 ± 1.11	145.30 ± 1.78	257.15 ± 3.18
	Experimental chicks	Male	43.99 ± 0.53	91.36 ± 1.22	151.96 ± 2.22	284.32 ± 4.18
		Female	42.15 ± 0.47	86.20 ± 1.07	138.28 ± 1.69	249.60 ± 3.49

Mean mass (grams) ± s.e.m. of control (n = 48) and experimental chicks (n = 50) on post-hatch days 1, 10, 16 and 25.

### Influence of periodic cooling on fear of novelty

We found a significantly higher latency to eat a novel food in experimental chicks than in control chicks, thus suggesting a stronger food neophobia in experimental chicks (Table 3). Except for a preliminary inhibitory phase, times spent eating during tests did not differ significantly between groups. No significant differences were observed between groups when chicks were exposed to a novel object (Table 3). Latencies to eat were significantly longer in the novel object test with an unfamiliar trough than in the habituation test with the familiar trough for both control (*z* = −3.63, *P* < 0.01) and experimental chicks (*z* = −3.65, *P* < 0.01). Times spent eating during tests were significantly longer in the habituation test than in the novel object test for both control (*z* = −3.18, *P* < 0.01) and experimental chicks (*z* = −2.67, *P* < 0.01). Tested individually in a novel environment (open-field test), experimental chicks showed stronger fear reactions associated with a stronger behavioural inhibition than did control chicks (Table 3). First step and distress call latencies were longer for experimental chicks than for control chicks. The open-field test commonly elicits first an acute fear response in response to isolation and novelty. This acute aversive response can be assessed by the duration of an initial freezing phase (duration spent immobile or latency of first step). Greater fear responses are also associated with reduced activity levels (calling, jumping or walking)^26^. In line with this, experimental chicks expressed fewer distress calls, fewer jumps against the wall and tended to cross fewer lines than control chicks.

**Table 3:** Neophobia of control and experimental chicks.

Neophobia Tests	Parameters Measured	Control chicks (n = 25)	Experimental chicks (n = 24)	*U*	*P-*values
**Habituation**	latency to eat (seconds)	63.5 ± 8.38	77.04 ± 9.45	360.50	0.22
	time spent eating (seconds)	42.16 ± 5.02	37.48 ± 5.24	264.50	0.50
**Novel food**	latency to eat (seconds)	18.79 ± 3.12	40.96 ± 3.90	402.00	0.04
	time spent eating (seconds)	31.48 ± 4.53	36.88 ± 4.75	252.00	0.33
**Novel Object**	latency to eat (seconds)	128.04 ± 55.36	141.88 ± 57.67	347.00	0.32
	time spent eating (seconds)	16.48 ± 28.56	17.29 ± 24.84	258.50	0.37

Parameters measured (mean ± s.e.m.) during the novel food, novel object and open-field tests with Mann-Whitney *U*-tests.

### Influence of periodic cooling on social behaviour and performance in a detour task

The social motivation of chicks did not differ significantly between groups. The results of the runway tests showed that neither latencies to join a familiar conspecific nor proportions of time spent in the social zone differed between groups (experimental chicks, n = 25 *vs*. control chicks, n = 24; latency to join the conspecific: 23.32 ± 4.34 *vs*. 27.00 ± 5.13 seconds, respectively, *U* = 1153.50, *P* = 0.74; proportion of time spent in the social zone: 0.74 ± 0.03 *vs*. 0.73 ± 0.03, respectively, *U* = 1267, *P* = 0.64). The total proportion of time spent close to conspecifics (familiar or not) in discrimination tests did not differ between groups (0.72 ± 0.04 *vs*. 0.78 ± 0.04, respectively, *U* = 256, *P* = 0.39). Within each group, chicks spent significantly more time in the zone close to the familiar conspecific than in the zone close to the non-familiar conspecific (experimental chicks, n = 25, 116.87 ± 16.07 *vs*. 36.67 ± 9.07 seconds, respectively, *z* = −2.94, *P* = 0.003; control chicks, n = 24, 123.08 ± 15.66 *vs*. 33.84 ± 8.58 seconds, respectively, *z* = −3.55, *P* = 0.0004). This clear preference showed that regardless of the treatment, chicks were able to discriminate conspecifics. Experimental chicks in a detour task with a familiar conspecific as goal stimulus, showed significantly higher latencies to go round the barrier than did control chicks (194.96 ± 35.46 *vs*. 116.16 ± 29.06 seconds, respectively, *U* = 413, *P* = 0.019).

### Faecal corticosterone levels

The fecal corticosterone metabolite levels did not differ significantly between groups (experimental chicks, n = 25 *vs*. control chicks, n = 24; 99.16 ± 4.38 *vs*. 100.58 ± 6.58 ng/g, respectively, *U* = 313, *P* = 0.80).

### Influence of periodic cooling on corticotropin-releasing factor expression in the amygdala

**Figure 1:**
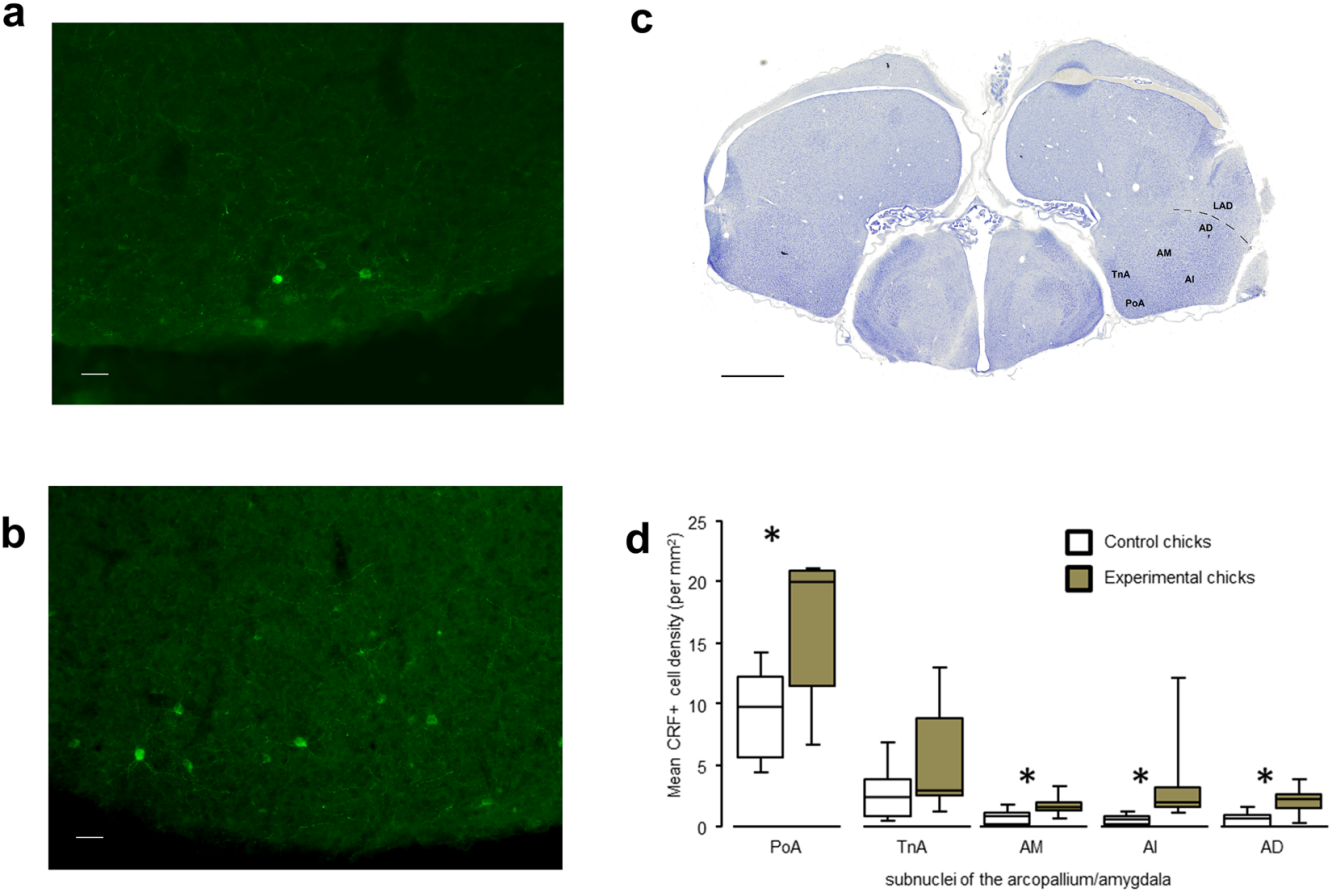
Representative photomicrographs of fluorescent corticotropin-releasing factor positive cells (CRF+) in the posterior pallial amygdala (PoA) of a control (**a**) and an experimental chick (**b**). Scale bar: 50 µm. (**c**) Cresyl Violet of a chick brain section illustrating the location of the posterior pallial amygdala (PoA), the Arcopallium dorsale (AD), the Arcopallium mediale (AM) and the Arcopallium intermedium (AI); LAD: lamina arcopallialis dorsalis. Scale bar: 1500 µm (**d**) Median and interquartile distribution ranges of the density of corticotropin-releasing factor positive cells (mean number/mm²) in the PoA, TnA, AM, AI and AD of control (N = 8) and experimental chicks (N = 7). *Mann-Whitney U-tests, *P* < 0.05.

The number of corticotropin-releasing factor immunopositive cells in the intermediate (*U* = 1.5, *P* = 0.002), medial (*U* = 8.5, *P* = 0.02), dorsal (*U* = 7.5, *P* = 0.01) and posterior amygdaloid (*U* = 11, *P* = 0.049) nuclei of the arcopallium/amygdala of experimental chicks was significantly higher than in those of control chicks. No significant differences were found in the TnA nuclei of the amygdala between groups (*U* = 18, *P* = 0.25; Fig. 1).

## Discussion

In this study, we provide the first evidence that incubation temperature may affect the neurobehavioural development of young birds. Chronic exposure to a suboptimal temperature delayed hatching, decreased growth rate and oriented the development of juveniles towards more fearful behavioural profiles. Chicks exposed chronically to a suboptimal temperature also showed higher expression of the corticotropin-releasing factor in several nuclei of the amygdala, a brain structure involved in fear-related behaviours. Our study thus sheds light on the underappreciated role of the incubation microclimate on the development of birds’ behaviour and its neurobiological correlates.

In accordance with previous findings^27^, we found that heart rates decreased approximately 50% after 1 hour of exposure to the suboptimal temperature of 27.2 °C. We also found that this exposure slowed embryonic development and lengthened incubation duration, traits well described in altricial or precocial bird species exposed to hypothermia^12,28,29^. Temperatures of birds’ allantoic fluid and embryonic heart rates show a linear relationship with a rapid decrease for both parameters when exposed to low temperatures^30^. This rapid adjustment of embryonic cardiac function is considered an adaptation that allows modification of oxygen supply under hypothermia^30^. Our treatment did not affect chicks’ mass at hatching or hatching success but did affect experimental chicks subsequently as during their first two weeks of life their mass was lower than that of control chicks. Because feeding motivation and faecal corticosterone metabolite levels did not differ between the two groups, this temporary alteration of growth could have been due to a higher catabolism of egg proteins and lipids in experimental than in control embryos or higher metabolic rates in experimental than in control chicks^31,32^. Our results are in accordance with experimental field studies. For instance, experimental cooling of the nest during incubation increased the duration of incubation and reduced growth rates in hatchlings of zebra finch (*Taeniopygia gutatta*)^28^, blue tits (*Cyanistes caeruleus*)^8^ and tree swallows (*Tachycineta bicolor*)^33^. Cooling of the nest can also reduce tarsus length after hatching in great tits (*Parus major*)^34^.

Our data revealed an effect of incubation temperature on the behavioural phenotypes of young chicks. In the open-field test, experimental chicks took significantly longer latency to take their first step than did control chicks, thus indicating a longer duration of the initial freezing phase. Duration of freezing is a parameter classically used to estimate birds’ and rodents’ fear in novel environments: the longer the freezing the greater their fearfulness^26^. Behavioural inhibition of experimental chicks was stronger as they expressed significantly less social reinstatement behaviours (latency of first distress call, number of calls, and number of jumps against the wall of the pen) than did controls. Experimental chicks’ longer latencies to eat a novel food also indicated their more pronounced reaction to novelty than that of controls. Similarly, exposure to low temperatures affects the behavioural profile of bearded dragons (*Pogona vitticeps*) that are more neophobic in the short-term than bearded dragons incubated in a warmer environment^35^. Food neophobia, object neophobia and fear of novel environments of domestic chicks and other avian species, were recently considered as independent behavioural traits^36,37^. This could explain why we did not observe differences between the groups for all the traits related to neophobia. In addition, the latencies observed in the novel object tests were close to the total duration (180 seconds) of the tests and a ceiling effect may have masked potential differences. The mechanisms causing behavioural differences related to egg incubation temperature remain unexplored in vertebrates.

Our data highlight the fact that prenatal exposure to suboptimal temperatures may drive specific neurodevelopmental plasticity in birds’ brain. Indeed, we observed a higher density of corticotropin-releasing factor cells in different nuclei of the amygdala of birds prenatally exposed to suboptimal temperature compared with controls. This effect depended apparently on the sub-region investigated, because no effect of treatment was observed in the TnA of the amygdala. Several functional studies demonstrated that the arcopallium/PoA complex, which includes the posterior pallial amygdala (PoA) + medial arcopallium (AM) + intermediate arcopallium (AI) + dorsal arcopallium (AD), is involved in the expression of fear-related behaviours in birds, whereas the TnA is primarily involved in sexual behaviour^38^. The distribution of corticotropin-releasing factor fibers in domestic chicken’s brain is comparable to that described for mammals, and the corticotropin-releasing factor is involved directly in the control of fear-related behaviour, in addition to having a role in the regulation of the hypothalamic-pituitary-adrenal (HPA) axis^39,40^. Corticotropin-releasing factor neurons in the hypothalamus begin to function approximately on embryonic day 14^41^, thus indicating that the system was functional during most of the treatment period (between days 12 and 19) of our study. Increased corticotropin-releasing factor levels decrease domestic chicks’ and Japanese quail’s (*Coturnix c. japonica*) expression of social reinstatement behaviours when chicks were separated from conspecifics^42^. Additional investigations are required to further our understanding of these mechanisms. One hypothesis would be that a higher central expression of the corticotropin-releasing factor in experimental chicks than in control chicks could explain their higher levels of neophobia (both of food and environment) and their lower expression of social reinstatement behaviours when isolated from conspecifics in a novel-environment. Social separation is known to be particularly stressful for gregarious birds^43^. Our results are more in line with a stronger emotional reaction to separation (freezing and behavioural inhibition) than with a lesser social motivation since no differences were observed between our two groups in situations where the test chicks were in the presence of their cage mate. In another precocial species, continuous incubation at a low temperature increases baseline corticosterone levels in wood duck (*Aix sponsa*) ducklings^44^. In our study, levels of fecal corticosterone metabolites did not differ between our two groups despite significant differences in density of corticotropin-releasing factor cells in different nuclei of the amygdala. These metabolites are good indicators of adrenocortical activity (pooled activity over 1–2 hours in birds)^19^. As we collected faecal samples after a resting period, we cannot exclude the hypothesis that chicks’ stress-induced concentrations of corticosterone, for example after novel environment tests, had differed between the two groups. In addition, as for mammals, the corticotropin-releasing factor can also intervene centrally in the regulation of fear-related behaviours^39,40^. Embryonic microclimate can also affect nestlings’ subsequent immunity^33,45^ but this study did not address this issue.

Chronic exposure of domestic chickens to a low incubation temperature can cause oxidative damage and changes in antioxidant pathways^46^. Moreover, central administration of the corticotropin-releasing factor in one-day-old chicks exposed to low temperature decreases oxidative damage in the brain^47^. Although we did not evaluate potential oxidative stress, temperature-induced oxidative damage has been reported for a large range of taxa (from invertebrates to birds)^48^. Thus, the increase in density of corticotropin-releasing factor cells that we observed could indicate a response of the central nervous system in order to cope with the challenge. To cope with hypothermia, this response mobilizes both appropriate automatic and neuroendocrine responses to the challenge and neuroprotective effects in brain tissues. Long-term effects on the oxidative profiles of chicks would be an alternative hypothesis to explain the behavioural differences we observed between our groups. Indeed, variations of birds’ expression of neophobia have recently been found to co-vary with the oxidative profiles of individuals^49^. Further investigations are required to test this hypothesis predicting a link between oxidative profile and neophobia. It is important to note that our experimental chicks that hatched on day 22 were not included in our analysis. We cannot therefore exclude the hypothesis that these chicks might have presented stronger effects of the treatment.

Latencies of experimental chicks to perform a detour task were longer than those of controls. In the scincid lizard (*Bassiana duperreyi*), cold-incubated individuals were less able than hot-incubated individuals to learn the location of a safe site^15^. In the honeybee (*Apis mellifera*), temperature during pupal development is related to learning and memory abilities^50^. However, in our study, the greater emotional reactivity of experimental chicks than of controls could explain their longer latencies to perform the detour. Additional investigations are required to determine whether incubation temperature would also alter the development of cognitive abilities. No differences were found between groups of chicks concerning their social motivation or their capacities to discriminate between familiar and unfamiliar conspecifics. Therefore, the differences observed between our two groups in the detour task could not be explained by differences in the motivation to join conspecific cage mates. In addition, this result indicates that incubation temperature may not alter the capacity of gregarious birds to recognize brood mates and to form cohesive flocks.

In conclusion, we provided evidence of strong neurobehavioural plasticity caused by experimental variations of embryonic microclimate. Under natural conditions, avian embryos may face much more drastic variations of nest temperature than those in our experimental setup. Female galliforms (e.g. waterfowl, grouse) are thought to take off-bouts to forage 2 to 5 times a day, the average off-bout lasting 1–2 hours^51–53^. Blood Pheasant (*Ithaginis cruentus*) females leave their nest once a day for nearly 7 hours every day throughout the 37-day incubation period and egg temperatures then cooled to below 10 °C for more than 3 hours^54^. Superb lyrebirds (*Menura superb*), a large passerine, also present a similar pattern of incubation^55^. Albeit commonly less drastic, large diurnal variations due to parental nest attentiveness are reported for a large range of avian taxa with either mono- or bi-parental parental care systems^5,56–58^. Environmental factors such as the presence of predators^59^, food resource availability^12^, weather^60^ and/or human disturbances^61^ can influence incubation behaviour but less attention has been focused on this route of developmental plasticity. Our results support the proposal that incubation temperature variation may be a source of significant inter-individual variability in the expression of fear-related behaviours. Our data identify important directions for future research to help understand how embryonic microclimate can program the ontogeny trajectory of young birds. According to the Predictive Adaptive Response hypothesis, early experience is a source of developmental plasticity that should be adaptive to the environmental conditions encountered later in life^62^. Suboptimal embryonic microclimates could predict future risky (e.g. presence of predators) or unfavourable environments (e.g. weather, resources) for the offspring. In the context of the Predictive Adaptive Response hypothesis, this early embryonic experience may thus be an important route for shaping adaptive physiological and behavioural responses. However, in the case of embryonic microclimate, sub-optimal temperatures were mostly found to alter offspring quality (e.g. mass, immunity)^6^ and this could indicate a physiological constraint rather than an adaptation. Birds’ neophobia appears to be plastic and more frequent in individuals experiencing high-risk environments^20^. Further field experiments are now required to determine the influence of temperature on developmental plasticity and the consequences in term of Darwinian fitness (survival, reproduction).

## Methods

### Ethics statement

All birds were maintained at the Experimental Unit PEAT of INRA (Nouzilly, France, license number B-37-175-1). All experiments were approved by the Ethics Committee for Animal Experimentation of Val de Loire, CEEA Vdl (reference number 02153.02) and were performed in accordance with the European Communities Council Directive 2010/63/UE. All animals were sold for rehoming at the end of the experiment.

### Eggs and temperature treatment

The layer breeder Novogen (SCEA JEGOU, France) provided 180 eggs from White Leghorn layers, which were separated into two groups with statistically equivalent masses: a control group of 90 eggs maintained at a constant temperature (37.8 ± 0.2 °C; 56% relative humidity) and a thermo-manipulated group (experimental) of 90 eggs exposed to periodic cooling (27.2 ± 0.2 °C; 56% relative humidity) daily and for a one-hour duration between days 12 and 19 of incubation (mean mass ± SEM; control eggs: 60.39 ± 0.27 g; experimental eggs: 60.62 ± 0.34 g; ANOVA, *F*_*1,178*_ = 0.28, *P* = 0.59). Because eggs incubated at low temperatures early in incubation have low hatching success^63^, we started the thermal treatment on incubation day 12 by following a protocol described previously^27^. Control group chicks were exposed to a thermal environment optimal for morpho-physiological development. Although a constant temperature of 37.8 °C does not represent the pattern that embryos may experience under natural conditions, this temperature is known to be the optimal thermal environment for hatchability, growth and the quality of domestic chicken layer hatchlings^64^. This temperature was chosen because of the lack of data on the effects of more natural thermal patterns (variation ranging between 20 °C and 40 °C)^5^ on phenotype outcomes. Until incubation day 12, both groups of eggs were maintained in the same incubator at the constant optimal temperature of 37.8 °C. From days 12 to 19, the group of thermally manipulated eggs was placed twice daily and for one hour in a similar incubator with equivalent hygrometry and automatic turning system but maintained at 27.2 ± 0.2 °C. The temperature of 27.2 °C was above the physiological zero temperature, 24–26 °C, at which development is suspended^5^. Domestic hens commonly leave their nest for 1–2 hours once a day to forage^65^. To mimic disrupted parental behaviour, the thermo-manipulated embryos were exposed daily to two distinct one-hour cooling periods, one in the morning and one in the afternoon. The treatment was applied between 8 AM and 7 PM at a different time each day to avoid habituation. On incubation day 20, all eggs were maintained at the constant optimal temperature. At that stage, embryos are in the process of actively piercing their egg membranes and when exposed to cool temperatures, they express “distress” vocalizations^66^. These behaviours are a potential non-controlled source of energetic expense. To control for the potential influence of additional movements due to the transfer of the eggs between the two adjacent incubators (only a few centimetres apart), the tray containing control eggs was taken out and replaced gently in the same incubator each time the treated eggs were taken out and placed gently in the adjacent incubator. The trays were equipped with individual holes maintaining eggs in a fixed position. Thus, no additional turning was produced when the eggs were transferred between incubators.

### Embryonic heart rate

Embryonic heart rates were measured using six digital egg monitors (Buddy™, Vetronic Services, UK). Twelve control eggs and 12 experimental eggs were specifically used for this measurement. Although the control eggs were not exposed to the cooling treatment, the heart rates of the control and experimental embryos were scored simultaneously. All of the 24 eggs were measured twice a day (before and after the 1 hour cooling treatment) on four consecutive days starting on incubation day 12. After day 15, the movements and size of embryos interfere with the infra-red light signal^67^. The monitors were placed in a room with a controlled temperature, and 4 series of 6 eggs were scored simultaneously. The experimenter recorded the number of beats per minute every 15 seconds for 90 seconds.

### Chicks and housing conditions

We kept 98 chicks (48 controls and 50 experimental) to evaluate their behavioural performances. They all hatched between incubation day 21 and 21.5. 94% of the control chicks hatched on day 21. The control chicks kept for behavioural and neurobiological investigations were taken randomly from this hatching peak. 24% of experimental chicks hatched on day 21 and 59.3% on day 21.5. The experimental chicks were taken from this population. 27% of the experimental chicks hatched on day 22. They were not included in this analysis in order to control age and post-hatching experience between and within groups. It ensured homogeneity between groups of chicks kept for behavioural characterization and those kept for neurobiological analysis. Each chick was identified with a numbered ring on its leg. The chicks were placed in pairs (from the same treatment) in wire-covered plastic cages (50 cm × 40 cm × 30 cm; length × width × height) with wood shavings on the floor. Cages were placed in two rooms and balanced for treatment. Chicks were maintained under an 11 h light/13 h dark cycle, with water and food available *ad libitum*. The sex of each chick was determined by comb size at 4 weeks of age (males have larger combs than females). The control group was composed of 33 females and 15 males, and the experimental group was composed of 25 females and 25 males. All the chicks were weighed on post-hatch days 1, 10, 16 and 25. Food intake was assessed for each pair of chicks over a 24-hour period on post-hatch days 11 and 23 days of age by weighing feeding troughs.

### Food and object neopobia

Behavioural responses to novel food or objects were assessed following protocols known to induce neophobia^36^. Each test was performed at the same age for all chicks (8 and 9 days old). Each test was run for 180 seconds. Because chicks become distressed when they are socially isolated, we tested cage mates together (n = 25 pairs of experimental chicks and n = 24 pairs of control chicks). Tests were performed in a different room but in an experimental cage that had the same features as the home cage. Testing commenced 90 minutes after the feeder had been removed from the home cage. Pairs were transported to the test room in a box and were deposited in an opaque enclosure within the test cage, opposite to the feeding trough. After 30 seconds, the enclosure was removed, and an unseen observer, blind to the treatment, recorded the behaviour of one marked focal chick of each pair. Focal chicks were chosen randomly when they were 2 days old and were tagged with a blue-coloured mark on their head (the focal birds were 12 female and 13 male experimental chicks and 16 female and 8 male controls). Latency to eat (the moment swallowing was observed) and time spent eating were recorded (in seconds). On post-hatch day 7, chicks were familiarized with the test cage and handling procedure. Their home cage feeding trough was placed in the test cage, filled with their usual food. This familiarization procedure was also used to control food motivation. Food neophobia was tested on posthatch day 8 with their feeding trough filled with cracked corn-wheat. Object neophobia was evaluated on post-hatch day 9, the novel object was an unfamiliar coloured feeder (yellow and green plastic instead of grey metal) containing their familiar food.

### Fear of novel environment

A novel environment test (open-field) was conducted on post-hatch days 14 and 15. The chicks were individually (n = 50 experimental chicks and n = 48 control chicks) placed in the middle of an open arena (open-field; 120 cm diameter) for 5 minutes. To assess their locomotor activity, two perpendicular lines were drawn in the arena, dividing the space into four equal parts. Latency of the first step, number of times a subject crossed a line, number of jumps against the wall, latency of distress calling and number of distress calls were recorded by an unseen experimenter, blind to the treatment.

### Social behaviour

To assess social motivation, a runway test was conducted with individual chicks on post-hatch days 16 and 17 (n = 50 experimental chicks and n = 48 control chicks). The apparatus was a straight 145 cm-long wire-mesh tunnel with a cage at the end of the tunnel where the subject’s cage mate was placed. The tunnel was divided into three zones of equal size: ‘non-social’ (far from the conspecific), ‘middle’ and ‘social’ (close to the conspecific) zones. Each pair of chicks was transferred to the test room. Then, the cage mate was placed in the cage, and the test chick was placed in the middle zone. One chick per pair was tested each day. The side with the social stimulus was counterbalanced between trials. An unseen experimenter, blind to the treatment, recorded the time spent in each zone for a 5-minute period (beginning after the subject had taken its first step) (in seconds). To evaluate the capacity to discriminate between two conspecifics (social discrimination), we used a simultaneous two-choice test paradigm following the protocol previously described^68^. All tagged chicks of each pair were tested on post-hatch day 18 (n = 25 experimental chicks and n = 24 control chicks). This test was performed in a rectangular arena measuring 80 cm × 60 cm × 31 cm (length x width x height). Two stimulus birds were placed in 27 cm × 20 cm × 31 cm compartments with a wire mesh top and front at the opposite sides of a starting point. One of these compartments contained the subject’s familiar cage-mate and the other compartment contained an unfamiliar chick subjected to the same treatment and of the same age as the test chick. A “close zone” was delineated in front of each cage (14 × 27 cm). Sides were counterbalanced between trials. After 30 seconds, the test bird was released, and the time spent in each close zone was recorded for a five-minute period.

### Detour task

This test was performed on post-hatch day 28 in the same arena as the social discrimination test except that a single wire-mesh goal compartment was placed at the opposite side of the starting point. All tagged chicks of each pair were tested (n = 25 experimental chicks and n = 24 control chicks). For each pair, the cage mate was placed in the goal compartment, whereas the test chick was placed 30 cm away in a U-shape barrier with a wire-mesh front wall and two opaque, vertical sidewalls. To solve the problem, the chick had to move away from its cage mate, lose sight of it and go round one end of the barrier. An unseen experimenter, blind to the treatment, evaluated the performances of chicks by the latency to make the detour (cross the barrier with the whole body) from the start location.

### Corticosterone assays

To evaluate chicks’ corticosterone levels, fecal corticosterone metabolite (FCM) concentrations were assayed. On post-hatch day 22, a mix of fresh fecal droppings was collected between 9:00 and 11:00 a.m. from each home cage. Each sample was homogenized and stored at −20 °C. From each sample, an aliquot (0.5 g) was extracted with 60% methanol and analysed by using a cortisone enzyme immunoassay (EIA) validated for chickens and previously described in detail^69,70^. Intra- and interassay coefficients of variation were below 10% and 15%, respectively.

### Corticotropin-releasing factor (CRF) Immunohistochemistry

On the day of hatching, 8 control chicks and 8 experimental chicks were deeply anaesthetized by an intraperitoneal injection of pentobarbital. Chicks were perfused transcardially via the left ventricle with 15 mL of 1% sodium nitrite in phosphate buffer saline, then with 70 mL of ice-cold fixative (4% paraformaldehyde solution in 0.1 M phosphate buffer, pH 7.4). The brain was removed from the skull, post-fixed by immersion for one hour in the same fixative and then cryoprotected in 20% sucrose for two days^71^. Brains were frozen in isopentane at −40 °C and stored at −80 °C. Brains were cut in 30 µm coronal sections on a cryostat, in a caudal to rostral direction. Seven series of alternate sections (with an equal distance of 180 µm to one another) were collected onto SuperFrost® glass slides. One series was stained with Cresyl Violet to provide accurate anatomical localization. The other series were stored at −80 °C for immunohistochemistry analysis. Immunohistochemistry of CRF was performed on 15 animals (eight control and seven experimental chicks). One experimental chick was excluded because of perfusion failure. After incubation for 20 minutes at −20 °C in acetone, sections were dried and rehydrated for 5 minutes in tris-buffered saline (TBS). Sections were then immersed for 1 to 2 hours in TBS-triton-azide (TBSTA)-BSA 1% at room temperature. Sections were incubated for 48 hours at 4 °C with a rabbit anti-CRF antibody (Peninsula, USA) diluted 1:2000 in TBS-BSA 1% and then rinsed in TBS and incubated for 2 hours at room temperature with donkey anti-rabbit-488 antibody (Jackson, UK) diluted 1:1000 in TBS-BSA 0.1%. For fluorescence detection of CRF, sections were rinsed in TBS and immersed in a Hoechst bath for 2 minutes (Hoechst 33258, 2 µg/mL in water; Invitrogen, USA). The sections were rinsed in two baths of water and one bath of TBS and then dried and cover-slipped under Fluoromount-G (SouthernBiotech, USA). Positive cell (CRF + cell) measurements were assessed using a motorized microscope (Axioskope 2; Zeiss, Germany) connected to the image analysis software Mercator® (Exploranova, La Rochelle, France) through a camera. With a 2.5 × objective, the nuclei of interest were outlined. We analyzed the expression of CRF in the following subnuclei of the arcopallium/amygdala: intermediate (IA), medial (MA), dorsal (DA), and posterior amygdaloid (PoA) and nucleus taeniae (TnA). Several functional studies have demonstrated that the arcopallium/PoA complex is involved in the expression of fear-related behaviours by birds^38^. The TnA has been primarily studied for involvement in sexual behaviour; therefore, in this study TnA was analysed as a control structure. The amygdala presented large quantities of CRF that is considered the primary neurotransmitter in the regulation of fear-related behaviours^39^. The brain regions were identified in accordance with the stereotaxic atlas of the brain of the chick^72^ and the revised nomenclature^73^. For each bird and each subnucleus of the arcopallium/amygdala, 5 to 8 sections were analyzed, depending on the extent of the region. CRF + fluorescent cells in the amygdala were counted manually inside the area of interest, in both hemispheres. The counts of the serial sections were averaged (total number of cells divided by the total area of all sections) to obtain a single value per brain region. The counter was blind to the treatment.

### Statistics

We compared the numbers of chicks hatched from fertile eggs by using a Chi-square test. We used a one-way ANOVA to compare the number of days of incubation between groups. The masses of chicks were compared by using a two-way repeated-measures ANOVA with treatment and sex as factors and a *post hoc* protected least significant difference Fisher test. Food intake was compared between groups using a one-way ANOVA. Even after transformation, the rest of the data were not normally distributed (Shapiro-Wilk test) and did not have the homogeneity of variances (Levene tests) required to apply parametric statistics. Wilcoxon tests with Monte-Carlo simulations were used, within groups, to compare the mean numbers of heart beats per minute before and after 1 hour of treatment, latencies to eat and times spent eating. Mann-Whitney *U*-tests with Monte-Carlo simulations were used for intergroup comparisons on all behavioural parameters recorded during neophobia tests (food, object, environment), during the detour task, on corticosterone metabolite concentrations and on the mean numbers of immunoreactive cells. To analyse social motivation (runway and discrimination), we compared the proportions of time spent in the social zone (time spent in the social zone/300 seconds) between groups using Mann-Whitney *U*-tests with Monte-Carlo simulations. We compared the time spent in the social discrimination test close to the familiar conspecific to the time spent close to the unfamiliar conspecific using Wilcoxon tests with Monte-Carlo simulations. Sex effects are mentioned only when significant. All analyses were performed with XLSTATS 2016.2 (Addinsoft), with significance accepted at *P* ≤ 0.05.

The datasets generated and analysed during the current study are available from Zenodo (10.5281/zenodo.1135998).
